# Dynamic Lactate Measurements Serve as an Effective Marker to Predict Short-Term Postoperative Outcomes in Patients Who Undergo Surgery for Acute Type A Aortic Dissection

**DOI:** 10.31083/RCM52906

**Published:** 2026-07-15

**Authors:** Zhonghua Sun

**Affiliations:** ^1^Discipline of Medical Radiation Science, Curtin School of Diagnostic and Therapeutic Sciences, Curtin University, Perth, WA 6845, Australia; ^2^Curtin Medical Research Institute (Curtin MRI), Curtin University, Perth, WA 6845, Australia

Type A acute aortic dissection (TAAAD) remains one of the most fatal cardiovascular emergencies, with an untreated mortality rate of approximately 1% per hour during the first 48 hours after symptom onset [1]. While emergent surgical repair is the standard of care, operative mortality still ranges from 10% to 15%, and outcomes worsen significantly when malperfusion syndromes complicate the presentation [2]. Multiple factors can influence the prognosis of patients with TAAAD, including advanced age, pre-existing comorbidities, such as myocardial infarction, the extent and location of the dissection tear, and the presence of malperfusion syndrome. Consequently, identifying robust prognostic factors is essential for risk stratification and for developing personalized postoperative management strategies for patients undergoing surgery for TAAAD.

Serum lactate has emerged as a clinically valuable biomarker that reflects the systemic metabolic consequences of tissue hypoperfusion, which affects one-third of patients with TAAAD [3]. Previous studies have demonstrated a clear association between elevated lactate levels and an increased risk of mortality in patients with TAAAD [4,5,6,7]. These findings suggest that preoperative lactate levels may reflect the baseline burden of malperfusion. In contrast, postoperative lactate trends may indicate the adequacy of surgical repair and the persistence of impaired perfusion, thereby contributing to adverse outcomes. However, most existing studies have been limited by a reliance on single-time-point lactate measurements. Meanwhile, evidence regarding the prognostic value of dynamic lactate changes for short-term mortality prediction in patients with TAAAD remains sparse. This research gap has been addressed by a recent study reported by Chen et al. [8].

In a recent issue of Reviews in Cardiovascular Medicine, Chen and colleagues [8] explored the relationship between dynamic lactate levels and short-term mortality in patients with TAAAD following surgery. In this single-center study, 178 patient records were reviewed, and 146 individuals were ultimately included: 101 in the survival group and 45 in the non-survivor group. All patients underwent preoperative assessment and classification using computed tomography angiography, and arterial lactate levels were measured by laboratory examinations at three time points: immediately postoperatively (IPO) upon admission to the intensive care unit (ICU), postoperative day 1 (POD1), and postoperative day 3 (POD3). The authors calculated lactate clearance (LC) as the percentage reduction in lactate from baseline on postoperative days 1 and 3.

The results revealed that elevated lactate levels at these intervals were strongly associated with short-term mortality (*p* < 0.001), whereas myocardial infarction was not an independent predictor (*p* = 0.319). Using repeated-measures analysis via generalized estimating equations, the investigators confirmed a significant difference in lactate levels between the survival and non-survival groups, with significantly higher levels in the non-survival group (*p* = 0.040). The optimal cut-off values for lactate elevation were 2.15 mmol/L, 7.85 mmol/L, and 1.75 mmol/L at the respective time points (*p* < 0.001 for all). For LC, the corresponding values were –218.91% for postoperative day 1 and 37.23% for postoperative day 3 (*p* = 0.004–0.090). Increased lactate levels were associated with a higher risk of mortality in both male and female patients.

Interestingly, both lactate and LC were strong predictors of short-term mortality in patients with TAAAD. Further analysis showed that lactate levels were significantly higher in patients aged >55 years than in those aged 45 years or younger (*p* < 0.001), and in patients with creatinine ≤98 µmol/L than in those with creatinine >98 µmol/L (*p* = 0.009). Meanwhile, LC was particularly relevant in patients older than 50 years compared with those aged 45 years or younger (*p* = 0.014), consistent with previous reports that reduced LC can lead to multiple organ failure and increased mortality in patients with sepsis, trauma, and respiratory failure [9,10].

**Fig. 1.**
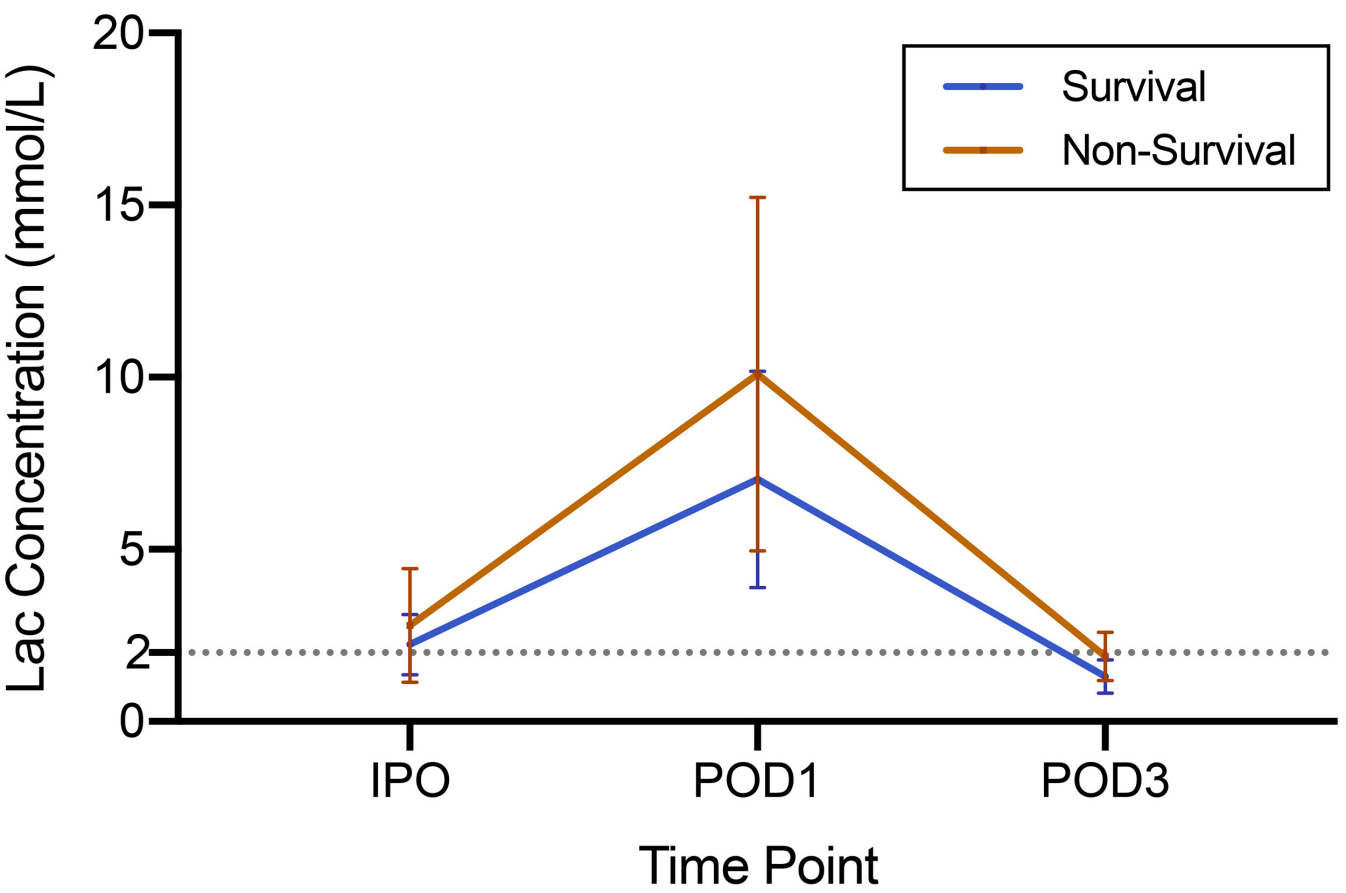
**Characteristics of lactate levels in survivors and non-survivors at different time points**. Data are presented as mean ± standard deviation. Reprinted with permission under open access from Chen et al*.* [8]. IPO, immediately postoperatively; POD1, postoperative day 1; POD3, postoperative day 3.

Two important findings from a recent study by Chen et al. [8] deserve further discussion. First, the authors provide compelling new evidence supporting the use of dynamic lactate levels to predict outcomes in patients with TAAAD following surgery. While elevated lactate concentrations have already been recognized as highly sensitive markers for outcome prediction in patients with TAAAD [5,6,7], most previous research has focused on a single time point, specifically the correlation between postoperative 24-hour lactate levels and short-term mortality in surgically treated TAAAD [11,12]. What distinguishes this study is the associated demonstration of superior predictive value of monitoring lactate over a 72-hour postoperative period, offering a broader window to capture dynamic changes and support risk assessments (Fig. 1, Ref. [8]).

**Fig. 2.**
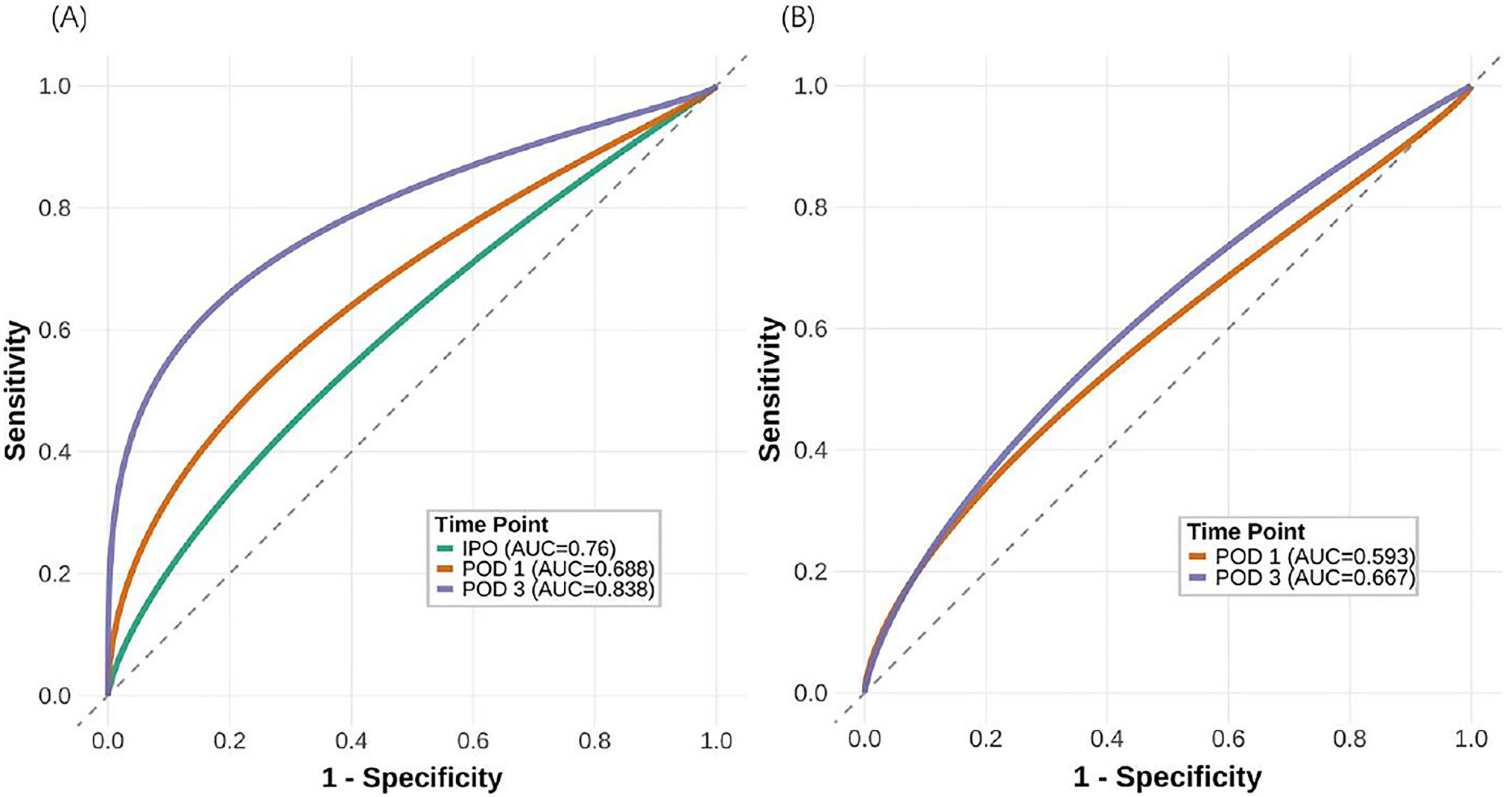
**Receiver operating characteristic (ROC) curves of lactate and lactate clearance (LC) for short-term mortality**. (A) ROC of multivariate logistic regression for the association between lactate and death immediately postoperatively (green) on postoperative day 1 (red), and on postoperative day 3 (blue). (B) ROC of multivariate logistic regression for the association between LC and death on postoperative day 1 (red) and postoperative day 3 (blue). Reprinted with permission under the open access from Chen et al. [8]. AUC, area under the curve.

Second, the study advances the field by establishing cut-off values for both lactate and LC to predict adverse outcomes following TAAAD surgery. Previous research has used a variety of mean values for preoperative and postoperative lactate levels, resulting in inconsistent findings across studies (**Supplementary Fig. 1**) [7]. In contrast, the findings by Chen et al. [8] provide a more objective approach to early postoperative risk stratification by clearly defining these cut-off values. A growing body of evidence links elevated lactate and reduced LC to adverse outcomes in patients undergoing surgery for TAAAD [12,13]; however, earlier studies have focused either on preoperative measurements or on the first 24 hours after surgery. This study extends those findings by showing that measurements obtained during the first 3 postoperative days are effective for predicting prognosis. The identified lactate cut-off value of >1.75 mmol/L is slightly lower than the 2.2 mmol/L measured at 6 hours after ICU admission reported by Gemelli et al. [5], likely reflecting differences in measurement timing and in the use of dynamic monitoring across studies. Wang et al. [12] reported dynamic changes in lactate levels measured at 0, 1, 6, 12, and 24 h after ICU admission, with lactate levels higher than 3.35 mmol/L at 12 h and 2.95 mmol/L at 24 h serving as reliable predictors of 30-day mortality (area under the curve (AUC) is 0.820 and 0.805, with corresponding sensitivity and specificity being 75.8% and 80.1%, 59.4% and 91.8%, respectively) (**Supplementary Fig. 2**). Additionally, the inclusion of LC as a predictive metric, with a cut-off value of ≤37.23% indicating an increased risk of short-term mortality, further deepens our understanding (Fig. 2, Ref. [8]). These findings offer important opportunities to improve risk assessment and outcome prediction in TAAAD surgery.

Although the study is limited by a relatively small sample size and the absence of preoperative lactate measurements, these findings still offer valuable insights into predicting postoperative outcomes in patients undergoing surgery for TAAAD. These data could also potentially contribute to improved patient care and more effective postoperative management after surgical repair of TAAAD. By highlighting these prognostic markers, research by Chen et al. [8] may help clinicians make more informed decisions and optimize postoperative management strategies. Owing to inconsistencies across existing studies regarding lactate thresholds for postoperative follow-up in patients with TAAAD, further research, particularly large, multicenter studies, is warranted to generate robust, generalizable evidence. Such work would facilitate the development of optimal follow-up protocols and support the incorporation of these strategies into routine clinical practice. Current clinical guidelines primarily focus on imaging surveillance and medical management for patients with TAAAD [14], and provide limited evidence or specific recommendations for the use of dynamic lactate measurements during postoperative follow-up.
