# Supplementary material for: Dynamic Lactate Measurements Serve as an Effective Marker to Predict Short-Term Postoperative Outcomes in Patients Who Undergo Surgery for Acute Type A Aortic Dissection
Source: Rev Cardiovasc Med. 2026 Jul 15;27(7):52906. doi: 10.31083/RCM52906 (PMC13419945; doi:10.31083/RCM52906)
Supplement: Supplementary file 1 [file 2153-8174-27-7-52906-s1.zip › 2153-8174-27-7-52906-s1.docx]

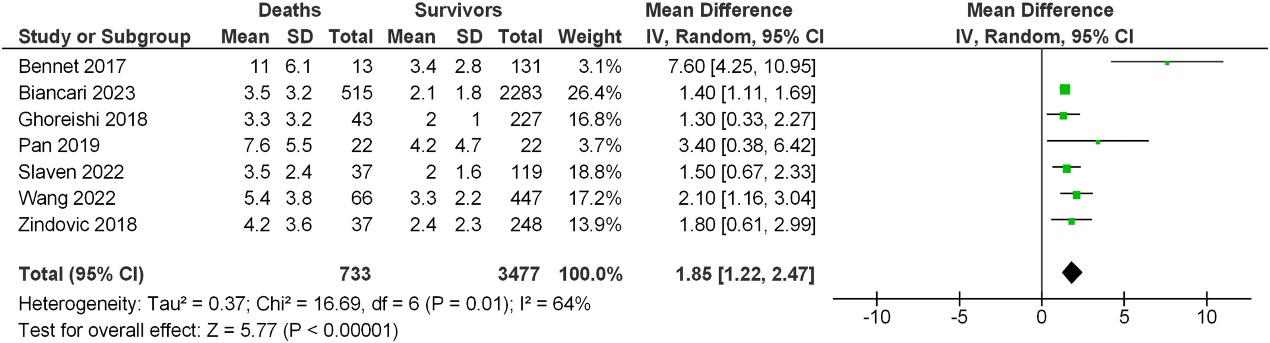


**Supplementary Fig. 1. Forest plot of the mean difference in preoperative arterial lactate concentrations in seven clinical studies that report on patients with type A aortic dissection.** Reprinted with permission under open access from Biancari et al. [1]. CI, confidence interval.


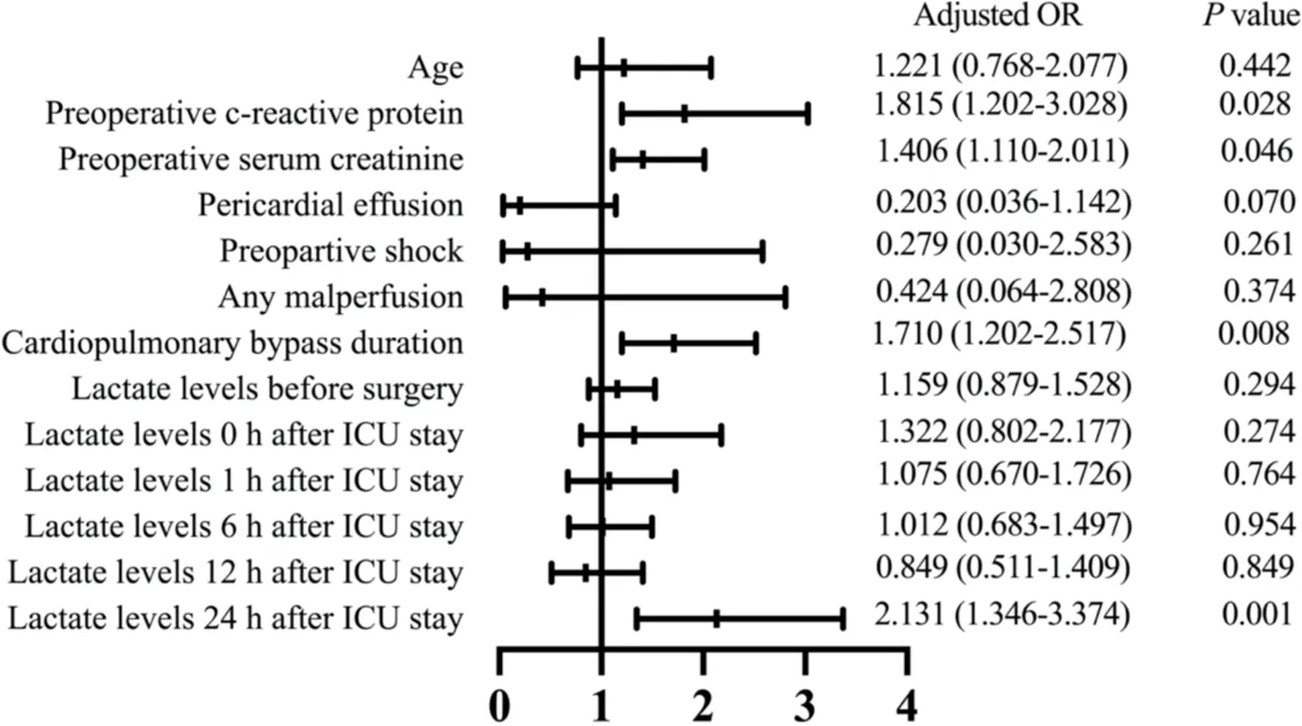


**Supplementary Fig. 2. Multivariate results that identify risk factors associated with 30-day mortality in patients who underwent type A acute aortic dissection (TAAAD) repair surgery.** OR, odds ratio; ICU, intensive care unit. Reprinted with permission under open access from Wang et al. [2].

## References:

[1] Biancari F, Nappi F, Gatti G, Perrotti A, Hervé A, Rosato S, et al. Preoperative arterial lactate and outcome after surgery for type A aortic dissection: The ERTAAD multicenter study. Heliyon. 2023; 9: e20702. <https://doi.org/10.1016/j.heliyon.2023.e20702>

[2] Wang Z, Li K, Xu J, Cheng X, Wang D. Construction of a lactate-related prognostic signature for predicting prognosis after surgical repair for acute type a aortic dissection. Frontiers in Physiology. 2022; 13: 1008869. <https://doi.org/10.3389/fphys.2022.1008869>
